# Temperature-dependent functional inversion of Ca_v_3.2 channels during repetitive activity

**DOI:** 10.1186/s13041-026-01327-w

**Published:** 2026-07-08

**Authors:** Kacper Pawlak, Petr Daněk, Bohumila Jurkovičová-Tarabová, Norbert Weiss

**Affiliations:** 1 Department of Pathophysiology, Third Faculty of Medicine, Charles University, Prague, Czech Republic; 2 Institute of Organic Chemistry and Biochemistry, Czech Academy of Sciences, Prague, Czech Republic; 3 Center of Biosciences, Institute of Molecular Physiology and Genetics, Slovak Academy of Sciences, Bratislava, Slovakia

**Keywords:** T-type channels, Ca_v_3.2, Channel gating, Temperature

## Abstract

Temperature strongly influences neuronal excitability and voltage-gated ion channel function. Ca_v_3.2 T-type calcium channels are highly expressed in dorsal root ganglion and trigeminal ganglion neurons, where they contribute to nociceptive signaling, cold hypersensitivity, and neuropathic pain. However, how temperature influences Ca_v_3.2 function during repetitive neuronal activity remains incompletely understood. Here, we investigated the effects of temperature on Ca_v_3.2 gating and calcium influx using whole-cell voltage-clamp recordings performed at 37 °C, 22 °C, and 13 °C. Cooling markedly reduced macroscopic current amplitude under conventional voltage-step protocols while profoundly slowing activation, inactivation, deactivation, and recovery from inactivation kinetics. In contrast, steady-state voltage dependence was comparatively less affected, revealing highly non-uniform temperature sensitivity across distinct gating transitions. During trains of action-potential-like waveforms delivered at 10–100 Hz, increasing stimulation frequency progressively inverted the temperature dependence of Ca_v_3.2-mediated calcium influx. Although cooling reduced peak current amplitude, it promoted sustained channel activity and enhanced cumulative calcium influx during high-frequency stimulation. This functional inversion resulted from disproportionate slowing of channel gating kinetics, particularly inactivation and deactivation. A kinetically slowed Ca_v_3.2 variant (V416R) reproduced key low-temperature kinetic features and markedly attenuated this inversion. Together, these findings identify a previously unrecognized temperature-dependent functional inversion of Ca_v_3.2 channels during repetitive activity with potentially important pathophysiological implications.

## Introduction

Voltage-gated T-type calcium channels are low-voltage-activated calcium channels that play central roles in regulating neuronal excitability, rhythmic firing, burst generation, and synaptic integration [1–3]. Among the three T-type channel isoforms, Ca_v_3.2 is broadly expressed in both the central and peripheral nervous systems, including dorsal root ganglion (DRG) and trigeminal ganglion (TG) neurons involved in somatosensory and nociceptive processing [4–6]. Through its ability to generate calcium influx near resting membrane potentials, Ca_v_3.2 contributes to rebound depolarization, repetitive firing, and amplification of excitatory inputs [7, 8]. Altered Ca_v_3.2 activity has been implicated in multiple pathological conditions, including epilepsy, primary aldosteronism, and multiple chronic pain conditions [9–11].

Temperature is a major determinant of ion channel function and neuronal excitability [12–16]. Changes in temperature can profoundly influence membrane biophysical properties, channel gating kinetics, and ion permeation. Previous studies have shown that temperature modifies the activity of multiple voltage-gated ion channels including voltage-gated calcium channels, often producing substantial changes in current amplitude and gating behavior [17–21]. In peripheral sensory neurons, cooling can alter firing activity and calcium signaling, including in neuronal populations not classically considered dedicated cold sensors [22–24]. However, despite the recognized temperature sensitivity of ion channels, the functional consequences of temperature on Ca_v_3.2 channel behavior remain incompletely understood [25].

Most previous studies examining temperature effects on voltage-gated calcium channels have relied on conventional voltage-step protocols. While such approaches provide important information regarding steady-state voltage dependence and peak current amplitude, they incompletely capture the dynamic behavior of ion channels during physiologically relevant patterns of repetitive activity. Because neuronal firing involves rapid and repetitive transitions between channel states, temperature-dependent alterations in gating kinetics may interact with firing frequency in complex and non-linear ways that are not readily predicted from conventional electrophysiological measurements. In particular, the relative temperature sensitivity of distinct Ca_v_3.2 gating transitions remains incompletely characterized [25]. Processes such as activation, inactivation, deactivation, and recovery from inactivation may exhibit markedly different thermal sensitivities, potentially reshaping channel behavior during repetitive stimulation. Such effects may be especially important in peripheral sensory neurons exposed to environmental cooling, where repetitive activity occurs under changing thermal conditions [22–24].

In the present study, we systematically investigated the impact of temperature on Ca_v_3.2 channel gating and calcium influx using whole-cell voltage-clamp recordings performed at 13 °C, 22 °C, and 37 °C. We combined conventional voltage-step protocols with action-potential-like repetitive stimulation paradigms to determine how temperature-dependent gating changes influence channel function during physiologically relevant activity patterns. We further examined a kinetically slowed Ca_v_3.2 variant (V416R) to directly assess the contribution of slowed gating transitions to temperature-dependent channel behavior.

Our results reveal that temperature exerts highly non-uniform effects on Ca_v_3.2 gating. While cooling strongly suppresses macroscopic current amplitude under conventional voltage-step conditions, repetitive activity uncovers a previously unrecognized temperature-dependent functional inversion whereby lower temperatures progressively enhance sustained Ca_v_3.2-mediated calcium influx during high-frequency stimulation. Mechanistically, this inversion emerges from disproportionate slowing of channel gating kinetics during cooling and can be largely occluded when Ca_v_3.2 channels are intrinsically shifted toward a slow-gating operational state. These findings identify a novel mechanism by which temperature dynamically reshapes Ca_v_3.2 channel function during repetitive activity and suggest potential implications for sensory neuron excitability and cold-evoked pain signaling.

## Materials and methods

### Plasmid cDNA constructs and site-directed mutagenesis

The Ca_v_3.2 V416R variant was generated by site-directed mutagenesis (GenScript) using the wild-type human Ca_v_3.2 construct (including exon 26) in pcDNA3.1 (kindly provided by Dr. Terrance Snutch) as a template. The fidelity of all constructs was confirmed by full-length sequencing of the coding region.

### Cell culture and heterologous expression

Human embryonic kidney tsA-201 cells were cultured in DMEM supplemented with 10% fetal bovine serum and 1% penicillin/streptomycin (all reagents from Invitrogen) and maintained under standard conditions at 37 °C in a humidified atmosphere with 5% CO_2_. Heterologous expression of Ca_v_3.2 channels was achieved by transfecting cells with 5 μg of plasmid cDNA encoding Ca_v_3.2 channel variants using the calcium/phosphate method.

### Patch clamp electrophysiology

Patch clamp recordings of T-type currents in tsA-201 cells expressing Ca_v_3.2 channel variants were performed 48 h after transfection in the whole-cell configuration. Recordings were conducted at 37 °C, 22 °C, and 13 °C using a single-channel temperature controller (Warner Instruments). The choice of 13 °C was made to probe channel behavior under a physiologically relevant range of cold temperatures, and within the range of temperatures that can be encountered in peripheral tissues under environmental cold exposure or local cooling conditions, where tissue temperature can drop substantially below 20 °C. The bath solution contained (in mM): 2 CaCl_2_, 165 choline-chloride, 1 MgCl_2_, 10 4-(2-hydroxyethyl)-1-piperazineethanesulfonic acid (HEPES) (pH 7.4 adjusted with CsOH). Patch pipettes were filled with a solution containing (in mM): 135 CsCl, 1 MgCl_2_, 10 EGTA, and 10 HEPES (pH 7.4 adjusted with CsOH), and had a resistance of 1.5–3.5 MΩ. Recordings were performed using an Axopatch 200B amplifier (Axon Instruments) and data acquisition and analysis were performed using pClamp 10.7 and Clampfit 10.7, respectively. Linear leak currents were corrected online. Currents were digitized at 10 kHz and low-pass filtered at 2 kHz.

The voltage dependence of activation was determined from peak T-type current amplitudes evoked by 150 ms depolarizing steps to various potentials, applied every 5 s from a holding potential of − 100 mV. Current–voltage (*I*-*V*) relationships were fitted with a modified Boltzmann equation:

$$I\left(V\right)=Gmax\frac{(V-Vrev)}{1+exp\frac{(V0.5-V)}{k}}$$

where *I*(*V*) is the peak current at command potential *V*, *G*_max_ is the maximal conductance, *V*_rev_ is the reversal potential, *V*_0.5_ is the half-activation potential, and k is the slope factor. The voltage dependence of the whole-cell calcium conductance was calculated as:

$$G\left(V\right)=\frac{Gmax}{1+exp\frac{(V0.5-V)}{k}}$$

Steady-state inactivation was assessed by measuring peak currents elicited by a 50 ms test pulse to − 20 mV following 5 s conditioning prepulses raging from − 120 to − 10 mV. Currents were normalized to the maximal response and plotted as a function of prepulse potential. Data were fitted with a two-state Boltzmann function:

$$I\left(V\right)=\frac{Imax}{1+exp\frac{(V-V0.5)}{k}}$$

where *I*_max_ is the maximal current and *V*_0.5_ is the half-inactivation potential.

Recovery from inactivation was assessed using a double-pulse protocol from a holding potential of − 100 mV. A 5 s depolarizing prepulse to 0 mV was used to fully inactivate channels, followed by a 50 ms test pulse to − 20 mV after variable recovery intervals (0.1 ms to 25.6 s) at − 100 mV. Peak test currents were normalized to the maximal response and plotted against interpulse interval. Data were fitted with a single-exponential function:

$$\frac{I}{Imax}=A*(1-exp\frac{-t}{\uptau })$$

where τ is the time constant of recovery from inactivation.

Calcium influx during action-potential-like activity was assessed by measuring the area under individual current transients (using a custom R script) elicited by 2 ms depolarizing steps to + 40 mV from a holding potential of − 80 mV, applied at 10, 20, 50, and 100 Hz.

Q10 values were calculated according to the equation:

$$Q10=\left(\frac{P2}{P1}\right)exp(\frac{10}{T2-T1})$$

where *P*_1_ and *P*_2_ represent the measured parameter values at temperatures *T*_1_ and *T*_2_, respectively.

## Statistics

Data are presented as mean ± SEM for *n* measurements. Statistical analyses were performed using GraphPad Prism 8. Significance was assessed using either Student’s t-test or one-way ANOVA followed by Dunnett’s post-hoc test. Differences were considered statistically significant at *p* ≤ 0.05.

## Results

### ***Temperature-dependent modulation of Ca***_***v***_***3.2 channel gating and macroscopic current output***

**Fig. 1:**
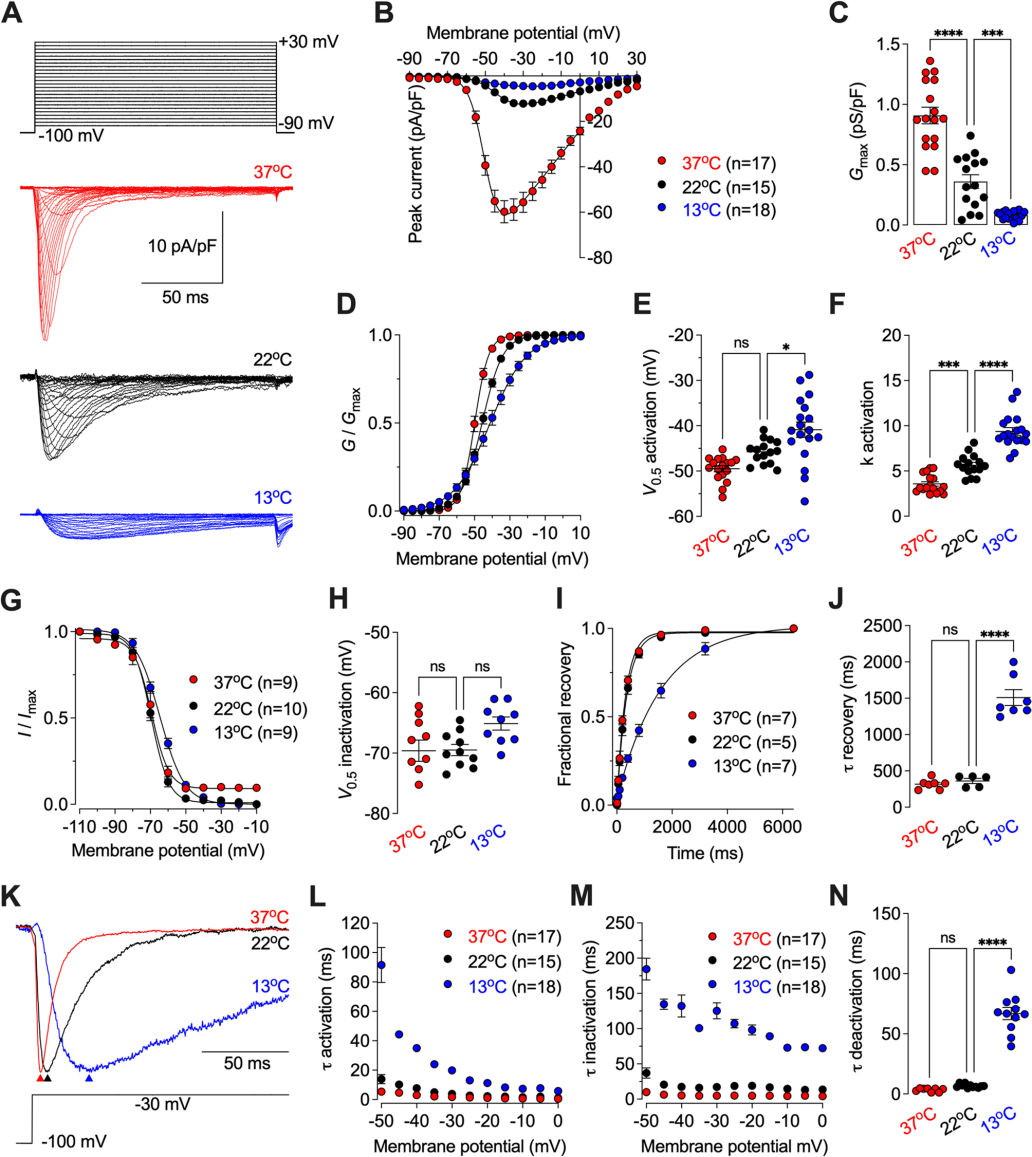
Temperature-dependent modulation of Ca_v_3.2 gating properties. **A** Representative whole-cell calcium currents recorded from Ca_v_3.2 channels in response to voltage-step protocols at 37 °C (red traces), 22 °C (black traces), and 13 °C (blue traces). Currents were evoked by depolarizing steps from a holding potential of − 100 mV. **B** Corresponding current–voltage (*I*-*V*) relationships obtained at each temperature and constructed from peak current amplitudes. **C** Maximal whole-cell conductance (*G*_max_) obtained from fitting *I*-*V* relationships with a modified Boltzmann equation. **D** Steady-state activation curves derived from conductance-voltage (*G*-*V*) relationships. **E** Corresponding half-activation voltage (*V*_0.5 act_) and **F** slope factor (*k*) obtained from fitting *G*-*V* curves with a Boltzmann function. **G** Steady-state inactivation curves obtained using standard pre-pulse protocols. **H** Corresponding half-inactivation voltage (*V*_0.5 inact_) obtained from fitting steady-state inactivation curves with a Boltzmann function. **I** Recovery from inactivation curves assessed using a paired-pulse protocol and plotted as normalized current recovery over time intervals. Data were fitted with an exponential function. **J** Corresponding time constant of recovery from inactivation (τ_rec_). **K** Superimposed, normalized Ca_v_3.2 current traces recorded at -30 mV at 37 °C (red trace), 22 °C (black trace), and 13 °C (blue trace), illustrating the temperature dependence of current activation and inactivation kinetics. **L** Corresponding time constant of current activation (τ_act_) and **M** inactivation (τ_inact_) plotted as a function of the command voltage, obtained from exponential fits of current rising and decay phases, respectively, during depolarizing voltage steps. **N** Time constant of deactivation (τ_deact_) measured from tail currents at -100 mV following 20 ms-long depolarizing steps to + 30 mV. Data are presented as mean ± SEM for (*n*) recoded cells, and statistical analysis was performed using an ANOVA test

To investigate the impact of temperature on Ca_v_3.2 channel function, we performed whole-cell voltage-clamp recordings at 13 °C, 22 °C, and 37 °C. Representative current traces evoked by depolarizing voltage steps from a holding potential of − 100 mV revealed marked temperature-dependent differences in Ca_v_3.2-mediated currents (Fig. 1A). Increasing temperature to 37 °C resulted in larger and more rapidly decaying currents, whereas lowering temperature to 13 °C reduced peak amplitude and substantially prolonged current time course. Consistent with these observations, current–voltage (*I*-*V*) relationships constructed from peak currents showed an overall increase in current density at 37 °C and a decrease at 13 °C across the tested voltage range (Fig. 1B). Quantification using a modified Boltzmann fit revealed a 2.5-fold increase of the maximal conductance (*G*_max_) at 37 °C (0.91 ± 0.07 pS/pF, *n* = 17; *p* < 0.0001) compared to 22 °C (0.36 ± 0.06 pS/pF, *n* = 15), and a 4.4-fold reduction at 13 °C (0.08 ± 0.01 pS/pF, *n* = 18; *p* = 0.0006) (Fig. 1C). Because macroscopic current amplitude reflects the combined contribution of multiple gating processes under voltage-clamp conditions, these changes indicate that temperature alters the overall functional output of Ca_v_3.2 channels. To determine whether these differences were associated with changes in voltage dependence, we analyzed conductance-voltage (*G*-*V*) relationships. Normalized activation curves exhibited temperature-dependent differences (Fig. 1D). The half-activation voltage (*V*_0.5 act_) was significantly shifted towards depolarized potentials at 13 °C (− 40.9 ± 1.7 mV, *n* = 18; *p* = 0.0115) compared to 22 °C (− 45.8 ± 0.7 mV, *n* = 15), whereas no significant difference was observed between 22 and 37 °C (− 49.5 ± 0.7 mV, *n* = 17;* p* = 0.0644) (Fig. 1E). In contrast, the slope factor (*k*) was significantly altered at both 13 °C (9.4 ± 0.4, *n* = 18; *p* < 0.0001) and 37 °C (3.6 ± 0.2, n = 17; *p* = 0.0003) relative to 22 °C (5.6 ± 0.3, *n* = 15) (Fig. 1F), indicating temperature-dependent changes in the steepness of voltage-dependent activation. Steady-state inactivation curves (Fig. 1G) revealed no significant effect of temperature on the half-inactivation voltage (*V*_0.5 inact_) (22 °C: − 69.5 ± 0.9 mV, *n* = 10; 37 °C: − 69.6 ± 1.8 mV, *n* = 9; 13 °C: − 65.1 ± 1.1 mV, *n* = 9) (Fig. 1H). In contrast, the slope factor was significantly increased at 13 °C (6.7 ± 0.3, *n* = 9; *p* < 0.0001) compared to 22 °C (4.2 ± 0.2, *n* = 7), whereas no significant difference was observed between 22 and 37 °C (3.6 ± 0.2, *n* = 10; *p* = 0.2241) (data not shown). These results indicate that temperature has little effect on the voltage dependence of steady-state inactivation but significantly alters its voltage sensitivity at lower temperature. In addition, inspection of the inactivation curves revealed that inactivation was incomplete at 37 °C, as evidenced by a small residual current persisting at depolarized pre-pulse potentials (Fig. 1G, red trace), whereas inactivation was more complete at 22 °C and 13 °C. This suggests that elevated temperature reduces the extent of steady-state inactivation without significantly altering its voltage dependence.

### Temperature strongly modulates gating kinetics across multiple transitions

We next examined the effect of temperature on gating kinetics. Recovery from inactivation, assessed using a paired-pulse protocol, was markedly slowed at lower temperatures and accelerated at higher temperatures (Fig. 1I). The time constant of recovery from inactivation (τ_rec_) was significantly increased at 13 °C (1510 ± 109 ms, *n* = 7; *p* < 0.0001) compared to 22 °C (362 ± 36 ms, *n* = 5), whereas no significant difference was observed between 22 and 37 °C (317 ± 26 ms, *n* = 7; *p* = 0.8788) (Fig. 1J), indicating strong temperature dependence of channel reavailability at low temperature. To visualize the combined effects of temperature on current kinetics, normalized Ca_v_3.2 current traces recorded at − 30 mV were superimposed (Fig. 1K). This comparison revealed that temperature reshapes the temporal profile of channel activity. Currents at 37 °C displayed rapid activation and inactivation, whereas currents at 13 °C exhibited markedly slower onset and prolonged decay. The time constant of activation (τ_act_) increased with decreasing temperature across the voltage range, indicating slower channel opening at 13 °C (Fig. 1L). For instance, at -30 mV, we observed a 5.1-fold increase of τ_act_ at 13 °C (19.8 ± 1.2 ms, *n* = 18; *p* < 0.0001) compared to 22 °C (3.9 ± 0.4 ms, *n* = 15), whereas a mild but not significant acceleration was observed between 22 and 37 °C (1.7 ± 0.2 ms, *n* = 17; *p* = 0.1142) (Fig. 1L). Similarly, the time constant of inactivation (τ_inact_) was significantly prolonged by 7.3-fold at 13 °C (125.3 ± 11.3 ms, *n* = 18; *p* < 0.0001) compared to 22 °C (17.2 ± 1.5 ms, *n* = 15), whereas a mild but not significant acceleration was observed at 37 °C (4.9 ± 0.4 ms, *n* = 17; *p* = 0.3855) (Fig. 1M), demonstrating that temperature accelerates entry into inactivated states. Finally, deactivation kinetics were assessed from tail currents measured at − 100 mV following brief depolarizing steps. The time constant of deactivation (T_deact_) was significantly increased at 13 °C (66.8 ± 5.1 ms, *n* = 11; *p* < 0.0001) compared to 22 °C (6.6 ± 0.5 ms, *n* = 11), whereas a mild but not significant decrease was observed at 37 °C (3.4 ± 0.5 ms, *n* = 8; *p* = 0.7276) (Fig. 1N), indicating prolonged open-state occupancy at lower temperatures.

### ***Non-uniform temperature sensitivity differentially affects Ca***_***v***_***3.2 gating transitions***

To further quantify the temperature dependence of Ca_v_3.2 channel gating, Q_10_ values were calculated separately between 13–22 °C and 22–37 °C for both steady-state and kinetic parameters (Table 1). Consistent with the overall increase in macroscopic current amplitude, *G*_max_ displayed marked apparent temperature sensitivity, with Q_10_ values of 5.20 between 13–22 °C and 1.85 between 22–37 °C, indicating a markedly stronger temperature dependence at lower temperatures. In contrast, steady-state voltage-dependent properties exhibited only limited temperature sensitivity. The voltage dependence of activation displayed relatively small apparent Q_10_ values for V_0.5 act_ (1.13 and 1.05 for 13–22 °C and 22–37 °C, respectively), while V_0.5 inact_ remained largely temperature-insensitive, with apparent Q_10_ values close to unity (1.07 and 1.00, respectively). Kinetic parameters, however, displayed markedly stronger and highly non-linear temperature dependence. The strongest effects were observed for deactivation and inactivation kinetics at low temperatures. Between 13 and 22 °C, τ_deact_, τ_inact_, τ_act_, and τ_rec_ exhibited apparent Q_10_ values of 0.08, 0.11, 0.16, and 0.20, respectively, whereas substantially weaker temperature sensitivity was observed between 22 and 37 °C (0.64, 0.43, 0.58, and 0.92, respectively).

**Table 1:** Temperature sensitivity of Ca_v_3.2 channel gating parameters

	G_max_	V_0.5 act_	V_0.5 inact_	τ_act_	τ_inact_	τ_deact_	τ_rec_
13–22 °C	5.20	1.13	1.07	0.16	0.11	0.08	0.20
22–37 °C	1.85	1.05	1.00	0.58	0.43	0.64	0.92

Summary of apparent Q10 values calculated for steady-state and kinetic parameters of Ca_v_3.2 channels between 13–22 °C and 22–37 °C. For kinetic parameters τ_act_, τ_inact_, τ_deact_, and τ_rec_), Q10 values below 1 indicate slowing of channel kinetics with decreasing temperature. Apparent Q10 values revealed markedly stronger temperature sensitivity for kinetic transitions compared to steady-state voltage-dependent parameters, particularly between 13 and 22 °C

Together, these findings demonstrate that temperature does not uniformly scale Ca_v_3.2 channel behavior, but instead differentially modulates individual gating transitions. In particular, kinetic processes exhibited substantially greater temperature sensitivity than steady-state voltage dependence, especially at lower temperatures, indicating that temperature primarily reshapes the temporal dynamics of Ca_v_3.2 gating rather than simply altering channel availability.

### ***Temperature-dependent Ca***_***v***_***3.2 gating differentially shapes calcium influx during repetitive action potential activity***

**Fig. 2:**
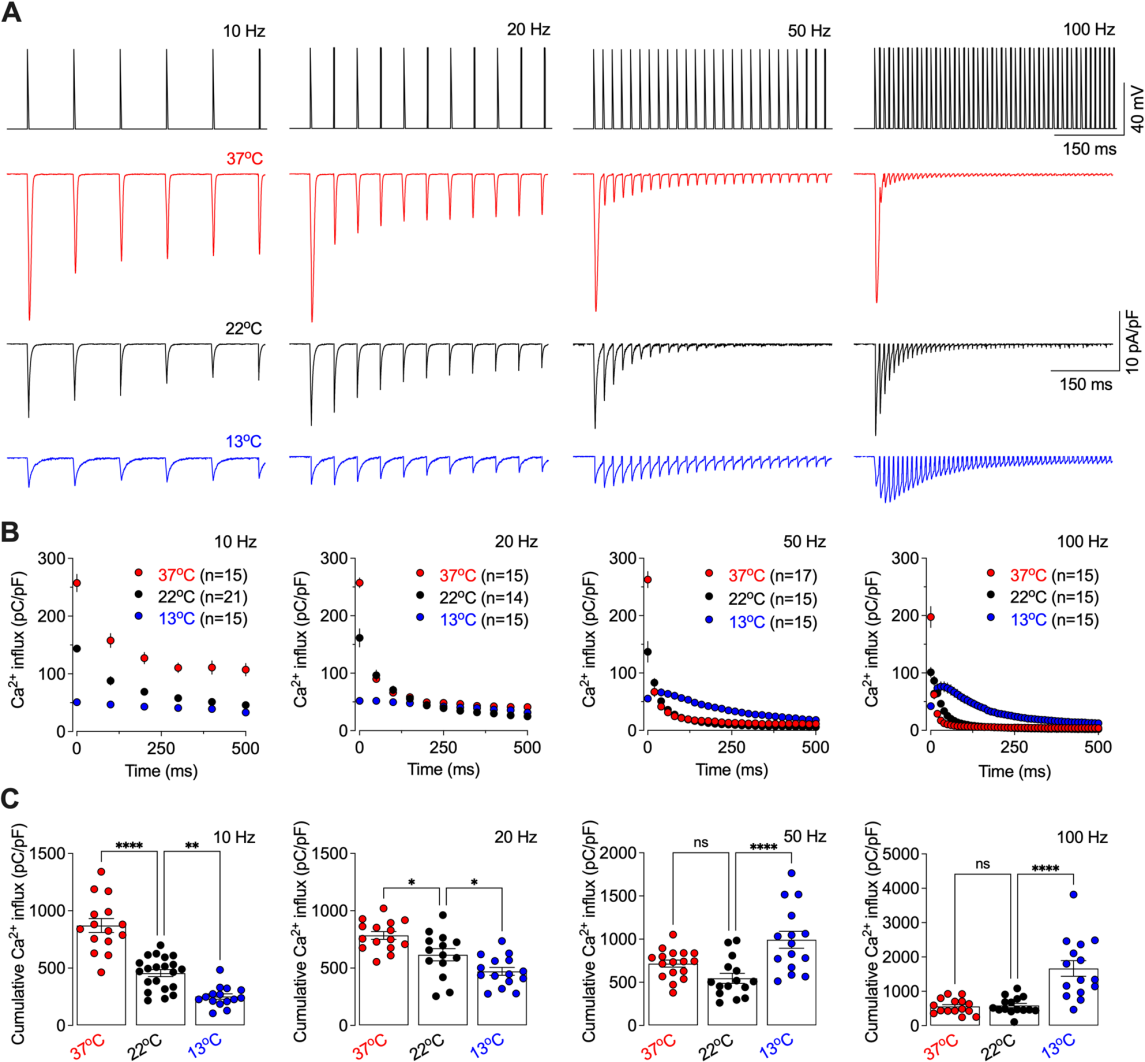
Frequency-dependent reversal of temperature effects on Ca_v_3.2-mediated calcium influx during repetitive action-potential-like stimulation. **A** Representative Ca_v_3.2 currents recorded in response to 500 ms-long trains of action-potential-like waveforms delivered at 10, 20, 50, and 100 Hz at 37 °C (red traces), 22 °C (black traces), and 13 °C (blue traces). **B** Corresponding calcium influx mediated by individual action-potential-like waveforms during the stimulation trains at each frequency and temperature condition. **C** Quantification of cumulative calcium influx measure during the 500 ms stimulation trains at each frequency and temperature condition. Data are presented as mean ± SEM for (*n*) recoded cells, and statistical analysis was performed using an ANOVA test

To determine how temperature-dependent changes in Ca_v_3.2 gating translate into channel function during physiologically relevant patterns of activity, we next applied 500 ms-long trains of action-potential-like waveforms delivered at frequencies ranging from 10 to 100 Hz (Fig. 2A). Representative recordings revealed marked frequency- and temperature-dependent differences in Ca_v_3.2-mediated calcium influx. At low stimulation frequency (10 Hz), currents recorded at 37 °C displayed the largest peak amplitudes throughout the train, whereas responses at 13 °C were markedly smaller (Fig. 2A and B). Quantification of cumulative calcium influx revealed a significant 1.9-fold increase at 37 °C (871 ± 61 pC/pF, *n* = 15; *p* < 0.0001) compared to 22 °C (456 ± 31 pC/pF, *n* = 21), whereas calcium influx was significantly reduced by 1.8-fold at 13 °C (252 ± 23 pC/pF, *n* = 15; *p* = 0.0011) (Fig. 2C). These findings indicate that the temperature-dependent modulation of Ca_v_3.2 functional output observed under conventional voltage-step protocols remains preserved during low-frequency repetitive activity. At 20 Hz, temperature-dependent differences in calcium influx became less pronounced. Although Ca_v_3.2 currents remained larger at 37 °C during the initial phase of stimulation, responses progressively converged across temperatures as the train progressed (Fig. 2A and B). Cumulative calcium influx remained significantly increased at 37 °C (785 ± 34 pC/pF, *n* = 15; *p* = 0.0109) and significantly reduced at 13 °C (471 ± 34 pC/pF, *n* = 15; *p* = 0.0297) compared to 22 °C (616 ± 53 pC/pF, *n* = 14) (Fig. 2C), indicating that the enhanced current amplitude observed at higher temperature becomes progressively counterbalanced by accelerated gating transitions during repetitive activity. In contrast, increasing stimulation frequency to 50 Hz profoundly altered the relative contribution of temperature to Ca_v_3.2-mediated calcium entry. While currents at 37 °C remained initially larger, they underwent rapid activity-dependent decline during the train, resulting in less sustained calcium influx than observed at 13 °C (Fig. 2A and B). At lower temperature, Ca_v_3.2 currents displayed slower decay and greater persistence throughout repetitive stimulation. Consequently, cumulative calcium influx at 13 °C was significantly increased by 1.8-fold (992 ± 98 pC/pF, *n* = 15; *p* < 0.0001) compared to 22 °C (546 ± 58 pC/pF, *n* = 15), whereas responses at 37 °C were not significantly different from those recorded at 22 °C (716 ± 41 pC/pF, *n* = 17; *p* = 0.1573) (Fig. 2C). These findings indicate that, at higher stimulation frequencies, the slowing of Ca_v_3.2 gating kinetics at lower temperature progressively outweighs the enhancement of peak current amplitude observed at elevated temperature. This inversion became even more pronounced at 100 Hz stimulation. Under these conditions, currents recorded at 37 °C rapidly decayed to near-baseline levels shortly after the onset of the train, whereas responses at 13 °C remained sustained over a substantially longer time window (Fig. 2A and B). Consequently, cumulative calcium influx at 13 °C showed a significant 2.8-fold increase (1663 ± 227 pC/pF, *n* = 15; *p* < 0.0001) compared to 22 °C (585 ± 63 pC/pF, *n* = 15) (Fig. 2C), whereas responses recorded at 37 °C (556 ± 57 pC/pF, *n* = 15) were not significantly different from those observed at 22 °C (*p* = 0.9844) (Fig. 2C). These findings demonstrate that, during high-frequency repetitive activity, the slowing of Ca_v_3.2 gating kinetics at lower temperature becomes the dominant determinant of sustained calcium influx.

Together, these findings demonstrate that the functional impact of temperature on Ca_v_3.2 channel activity is strongly frequency-dependent. Whereas elevated temperature enhances calcium influx during low-frequency stimulation, lower temperature promotes sustained Ca_v_3.2 activity during high-frequency repetitive firing. These observations indicate that temperature-dependent slowing of gating kinetics, particularly inactivation and deactivation, can outweigh the reduction in peak current amplitude during repetitive activity, thereby reshaping the net calcium influx carried by Ca_v_3.2 channels under physiologically relevant firing patterns and revealing a frequency-dependent reversal of temperature effects on Ca_v_3.2-mediated calcium influx.

### ***A Ca***_***v***_***3.2 variant with slowed gating kinetics reproduces key low-temperature kinetic features***

**Fig. 3:**
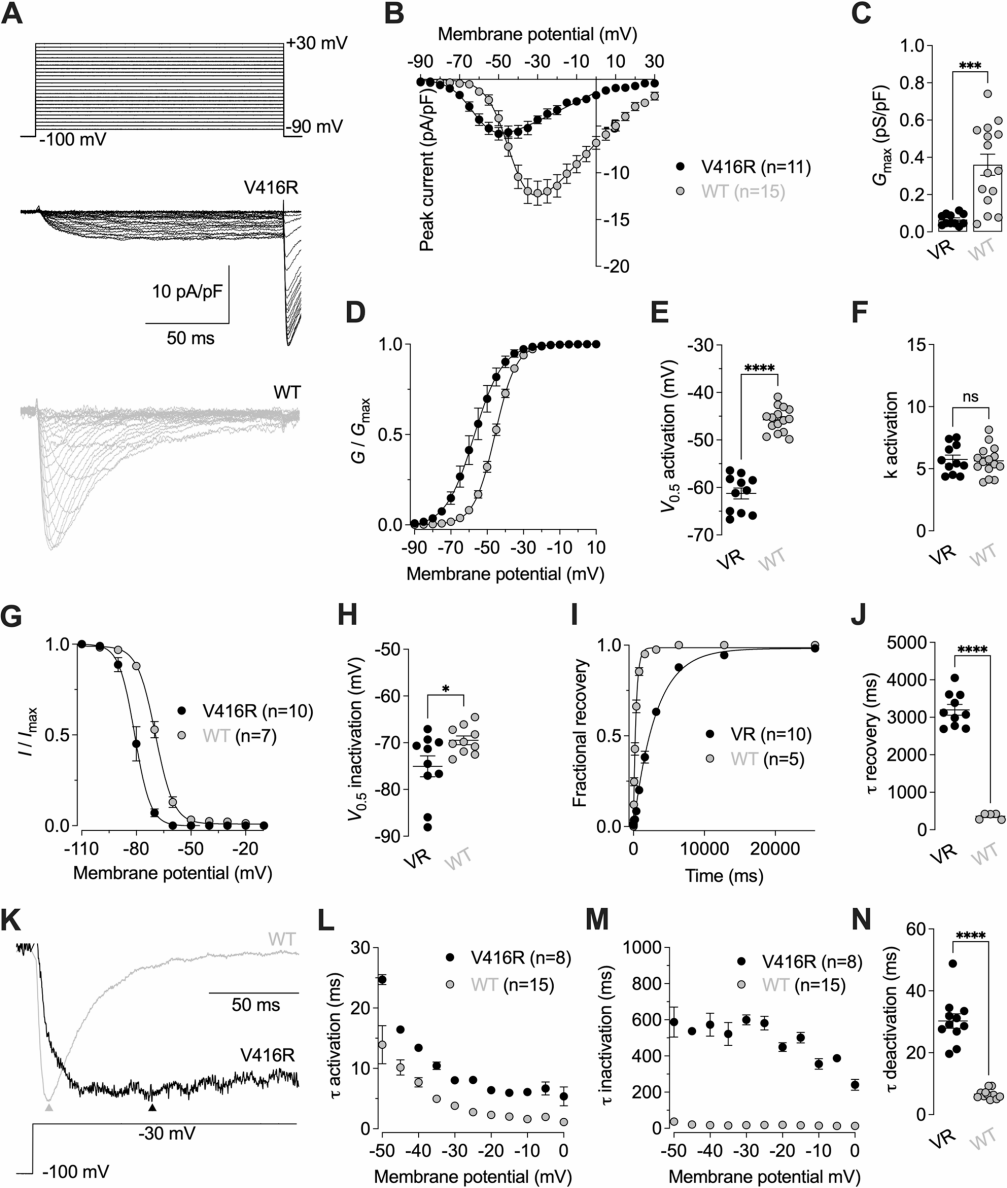
A Ca_v_3.2 variant with slowed gating kinetics reproduces key low-temperature kinetic features. **A** Representative whole-cell calcium currents recorded from V416R channels (black traces) in response to depolarizing voltage-step protocols from a holding potential of − 100 mV at 22 °C. Representative WT Ca_v_3.2 current recorded under identical conditions are shown in light gray for comparison. **B** Corresponding current–voltage (*I*-*V*) relationships constructed from peak current amplitudes. **C** Maximal whole-cell conductance (*G*_max_) obtained from fitting *I*-*V* relationships with a modified Boltzmann equation. **D** Normalized steady-state activation curves derived from conductance-voltage (*G*-*V*) relationships. **E** Corresponding half-activation voltage (*V*_0.5 act_) obtained from Boltzmann fits of activation curves. **F** Corresponding slope factor (k) of activation. **G** Steady-state inactivation curves obtained using standard pre-pulse protocols. **H** Corresponding half-inactivation voltage (*V*_0.5 inact_) obtained from Boltzmann fits of steady-state inactivation curves. **I** Recovery from inactivation curves assessed using a paired-pulse protocol and plotted as normalized current recovery over time intervals. Data were fitted with an exponential function. **J** Corresponding time constant of recovery from inactivation (τ_rec_). **K** Superimposed normalized current traces recorded at -30 mV from V416R (black trace) and WT channels (light gray trace), illustrating the effects of the mutation on current activation and inactivation kinetics. **L** Time constant of activation (τ_act_) plotted as a function of membrane potential and obtained from exponential fits of the current rising phase during depolarizing voltage steps. **M** Time constant of inactivation (τ_inact_) plotted as a function of membrane potential and obtained from exponential fits of current decay during depolarizing voltage steps. **N** Time constant of deactivation (τ_deact_) measured from tail currents recorded at − 100 mV following 20 ms-long depolarizing steps to + 30 mV. WT data shown for comparison correspond to the same 22 oC dataset presented in Fig. 1. Data are presented as mean ± SEM for (*n*) recorded cells, and statistical analysis was performed using unpaired Student’s t-tests

To further determine whether slowing Ca_v_3.2 gating transitions is sufficient to reproduce key aspects of the low-temperature phenotype, we generated a rationally engineered Ca_v_3.2 variant carrying the V416R substitution within the sixth transmembrane segment of domain I of the channel (IS6). This design was inspired by disease-associated arginine substitutions at the homologous position in high-voltage-activated calcium channels [26, 27], which are known to profoundly alter channel gating. We therefore hypothesized that introducing an arginine residue at this position in Ca_v_3.2 would bias the channel toward a slow-gating operational state. Representative current traces recorded at 22 °C revealed markedly slower current kinetics in V416R channels compared to WT Ca_v_3.2 (Fig. 3A). Consistent with this observation, current–voltage relationships showed reduced peak current density in V416R channels across the tested voltage range (Fig. 3B). Quantification using modified Boltzmann fits revealed a significant reduction in maximal conductance (*G*_max_) in V416R channels (0.07 ± 0.01 pS/pF, *n* = 11; *p* = 0.0002) compared to WT channels (0.36 ± 0.06 pS/pF, *n* = 15) (Fig. 3C). Analysis of voltage-dependent activation revealed a hyperpolarized shift of the activation curve in V416R channels (Fig. 3D). Accordingly, *V*_0.5 act_ was significantly shifted (*p* < 0.0001) from − 45.8 ± 0.7 mV (*n* = 15) in WT channels to − 61.3 ± 1.2 mV (*n* = 11) in V416R channels (Fig. 3E). In contrast, the slope factor of activation was not significantly altered between conditions (Fig. 3F). Steady-state inactivation properties were comparatively less affected by the mutation (Fig. 3G). Although *V*_0.5 inact_ displayed a modest but significant hyperpolarizing shift (*p* = 0.0336) from − 69.5 ± 0.9 mV (*n* = 10) in WT channels to − 75.1 ± 2.2 mV (*n* = 10) in V416R channels (Fig. 3H), the overall voltage dependence of inactivation remained relatively preserved. In contrast, kinetic analysis revealed pronounced slowing of Ca_v_3.2 gating transitions in V416R channels. Recovery from inactivation was markedly delayed in V416R channels compared to WT Ca_v_3.2 (Fig. 3I). The time constant of recovery (τ_rec_) was significantly increased (*p* < 0.0001) from 361 ± 36 ms in WT channels to 3199 ± 144 ms in V416R channels (Fig. 3J), indicating substantially delayed channel reavailability following inactivation. To directly visualize the effects of the mutation on current kinetics, normalized current traces recorded at − 30 mV were superimposed (Fig. 3K). Compared to WT Ca_v_3.2 currents, V416R channels displayed markedly slower activation and prolonged current decay. Consistent with these observations, the time constant of activation (τ_act_) was substantially increased across the tested voltage range in V416R channels (Fig. 3L). At − 30 mV, τ_act_ increased from 3.8 ± 0.4 ms (*n* = 15) in WT channels to 8.8 ± 0.6 ms (*n* = 8) in V416R channels (*p* < 0.0001). Similarly, the time constant of inactivation (τ_inact_) was markedly prolonged in V416R channels across the voltage range (Fig. 3M), indicating slower entry into inactivated states. At − 30 mV, τ_inact_ increased from 17.2 ± 1.5 ms (*n* = 15) in WT channels to 599.3 ± 27.3 ms (*n* = 8) in V416R channels (*p* < 0.0001). Finally, deactivation kinetics at − 100 mV were also profoundly slowed in V416R channels. The time constant of deactivation (τ_deact_) increased significantly (*p* < 0.0001) from 6.6 ± 0.5 ms (*n* = 11) in WT channels to 30.3 ± 2.3 ms (*n* = 11) in V416R channels (Fig. 3N), indicating prolonged channel open-state occupancy following repolarization.

Together, these findings demonstrate that the V416R substitution broadly slows Ca_v_3.2 gating kinetics while producing comparatively modest effects on steady-state voltage dependence. Notably, the kinetic phenotype of V416R channels closely resembles the slowing of gating transitions observed in WT Ca_v_3.2 channels at low temperature, suggesting that slowed gating kinetics represent a key determinant of the enhanced sustained calcium influx observed during high-frequency repetitive activity under cooling conditions.

### Intrinsic slowing of Cav3.2 gating kinetics reduces the temperature dependence of calcium influx during repetitive activity

**Fig. 4:**
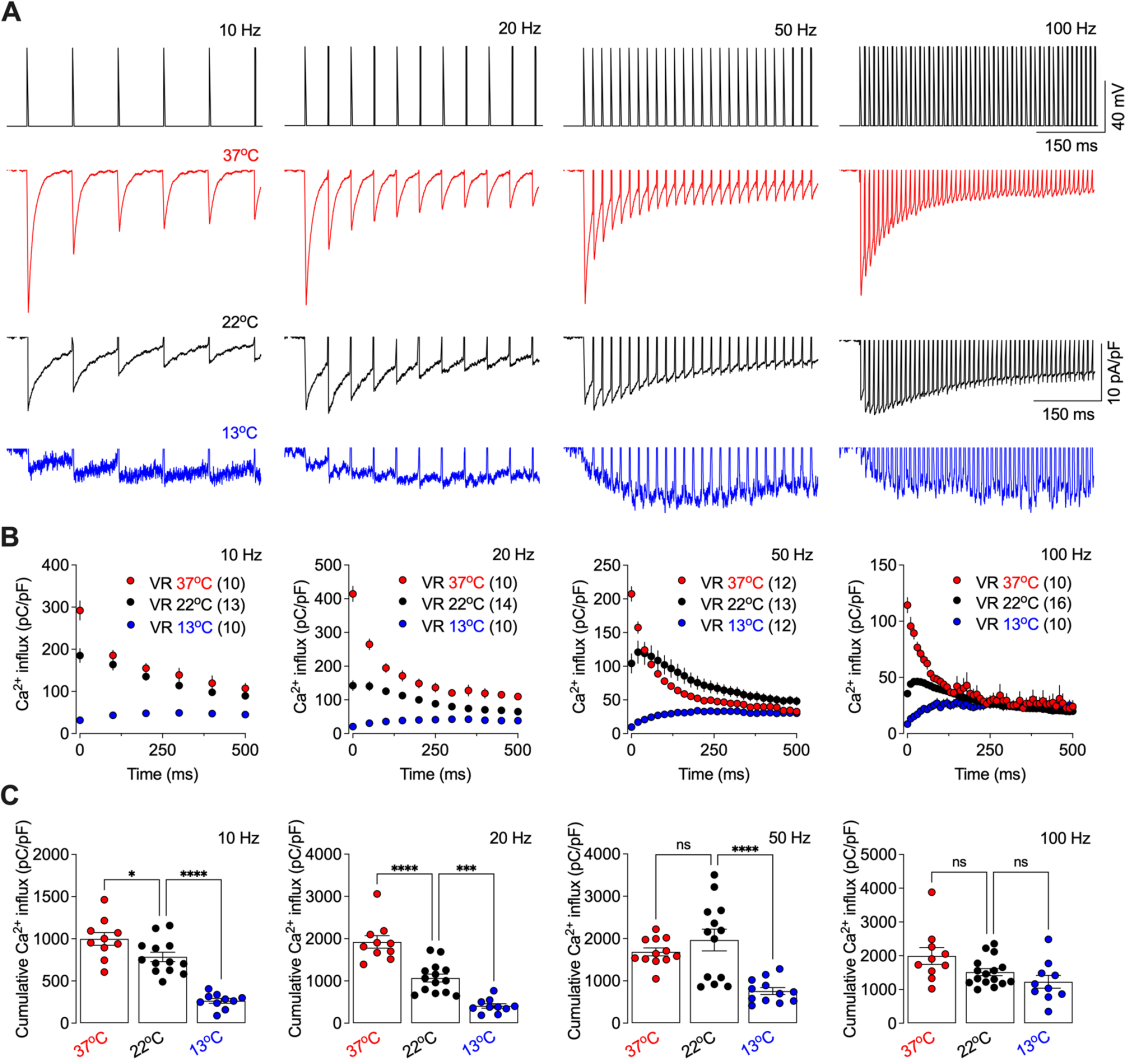
Temperature-dependent calcium influx mediated by the Ca_v_3.2 V416R mutant during repetitive action-potential-like stimulation. **A** Representative Ca_v_3.2 V416R currents recorded during 500 ms-long trains of action-potential-like waveforms delivered at 10, 20, 50, and 100 Hz at 37 °C (red traces), 22 °C (black traces), and 13 °C (blue traces). **B** Corresponding calcium influx mediated by each individual spike during the train. **C** Quantification of cumulative calcium influx during the train at each stimulation frequency. Data are presented as mean ± SEM for (*n*) recorded cells. Statistical analysis was performed using ANOVA

To determine whether intrinsically slowed Ca_v_3.2 gating kinetics alter the temperature dependence of calcium influx during repetitive firing, we next examined the V416R variant using the same action-potential-like stimulation paradigms applied to WT Ca_v_3.2 channels. Trains of action-potential-like waveforms were delivered for 500 ms at 10, 20, 50, and 100 Hz at 37 °C, 22 °C, and 13 °C (Fig. 4A). Representative current traces revealed distinct temperature- and frequency-dependent changes in V416R channel behavior during repetitive stimulation (Fig. 4A and B). At 37 °C, V416R-mediated calcium influx progressively declined throughout the train at all stimulation frequencies, although the rate of current decay appeared slower than in WT Ca_v_3.2 channels (Fig. 2A and B). At 22 °C, calcium influx also declined during low-frequency stimulation (10 Hz and 20 Hz), whereas higher stimulation frequencies (50 Hz and 100 Hz) produced an initial facilitation-like increase in calcium influx early in the train, followed by a progressive decline. In contrast, at 13 °C, calcium influx progressively increased throughout repetitive stimulation, with the magnitude of this potentiation becoming more pronounced as stimulation frequency increased (Fig. 4B). At 10 Hz stimulation, cumulative calcium influx remained highest at 37 °C (999 ± 75 pC/pF, *n* = 10), intermediate at 22 °C (786 ± 56 pC/pF, *n* = 13), and lowest at 13 °C (264 ± 28 pC/pF, *n* = 10), with significant differences observed between both 37 °C and 22 °C (*p* = 0.0229), and between 13 and 22 °C (*p* < 0.0001) (Fig. 4C). Similarly, at 20 Hz, cumulative calcium influx remained significantly increased at 37 °C (1922 ± 148 pC/pF, *n* = 10; *p* < 0.0001) and significantly reduced at 13 °C (408 ± 53 pC/pF, *n* = 10; *p* = 0.0002) compared to 22 °C (1068 ± 95 pC/pF, *n* = 14) (Fig. 4C). Thus, unlike WT Ca_v_3.2 channels, V416R channels retained the conventional temperature dependence of calcium influx at low and intermediate stimulation frequencies. At higher stimulation frequencies, however, temperature-dependent differences became progressively attenuated. At 50 Hz, cumulative calcium influx at 37 °C (1685 ± 92 pC/pF, *n* = 12; *p* = 0.4090) was no longer significantly different from 22 (1964 ± 256 pC/pF, *n* = 13), whereas responses at 13 °C remained significantly reduced (760 ± 80 pC/pF, *n* = 12; *p* < 0.0001) (Fig. 4C). At 100 Hz, temperature-dependent differences in cumulative calcium influx were further reduced, reaching 1993 ± 250 pC/pF at 37 °C, 1515 ± 104 pC/pF at 22 °C, and 1229 ± 189 pC/pF at 13 °C (Fig. 4C). Although cumulative calcium influx still tended to decrease with lowering temperature, no significant differences were observed between either 37 °C and 22 °C (*p* = 0.0962) or between 13 and 22 °C (*p* = 0.3964).

Together, these findings demonstrate that intrinsically slowed Ca_v_3.2 gating kinetics fundamentally reshape the temperature dependence of calcium influx during repetitive activity. In contrast to WT Ca_v_3.2 channels, in which cooling progressively shifted channel behavior toward sustained calcium influx during high-frequency stimulation, V416R channels displayed persistent calcium entry across stimulation conditions with substantially attenuated temperature-dependent functional inversion. At very high stimulation frequencies, repetitive activity progressively drove V416R channels toward a convergent functional state across temperatures, thereby reducing the overall impact of temperature on cumulative calcium influx. These results suggest that the temperature-dependent inversion observed in WT Ca_v_3.2 channels emerges from progressive slowing of gating transitions during cooling and can be largely occluded once the channel is intrinsically stabilized in a slow-gating operational state.

## Discussion

The present study demonstrates that temperature exerts complex and highly non-uniform effects on Ca_v_3.2 channel gating and function. While cooling strongly reduced macroscopic current amplitude under conventional voltage-step protocols, repetitive action-potential-like activity revealed an unexpected functional inversion whereby lower temperatures progressively enhanced sustained calcium influx during high-frequency stimulation. Mechanistically, this inversion emerged from disproportionate slowing of Ca_v_3.2 gating kinetics, particularly inactivation and deactivation, which progressively outweighed the reduction in peak current amplitude during repetitive activity. Furthermore, introduction of the kinetically slowed V416R variant largely occluded this temperature-dependent inversion, supporting the conclusion that slowed gating transitions represent a key determinant of the enhanced sustained calcium influx observed during cooling conditions.

Temperature is well known to modulate voltage-gated ion channel activity [17–21], yet its effects are often interpreted primarily in terms of current amplitude or generalized acceleration of channel kinetics. Previous work by Iftinca et al. [25] demonstrated that increasing temperature from room temperature to physiological temperature enhances Ca_v_3.2 current amplitude and accelerates channel gating kinetics. Our findings are broadly consistent with these observations, as we likewise observed larger currents together with faster activation and inactivation kinetics at 37 °C compared with 22 °C. Iftinca et al. also reported modest temperature-dependent effects on the voltage dependence of activation, recovery from inactivation, and deactivation kinetics. Although we observed similar trends, these effects did not reach statistical significance in our experiments. Such differences may reflect the use of rat Ca_v_3.2 channels in the study of Iftinca et al. versus human Ca_v_3.2 channels in the present work, as well as other methodological differences. Importantly, the study by Iftinca et al. was limited to comparisons between room and physiological temperatures and therefore did not address the effects of cooling on Ca_v_3.2 channel function. By extending the analysis to 13 °C, we demonstrate that cooling produces a disproportionate slowing of specific gating transitions, particularly inactivation and deactivation, thereby revealing a striking non-linearity in the temperature dependence of Ca_v_3.2 channel behavior. Steady-state voltage dependence was only modestly affected by temperature, whereas kinetic transitions displayed substantially stronger temperature sensitivity, particularly between 13 and 22 °C. Among the gating processes examined, deactivation and inactivation exhibited the strongest apparent temperature sensitivity, indicating that cooling profoundly alters the temporal dynamics of Ca_v_3.2 channel behavior rather than simply reducing channel availability. These observations are consistent with the notion that distinct conformational transitions within voltage-gated calcium channels possess different energetic barriers and therefore distinct thermal sensitivities. It is important to note that some of the temperature-dependent effects observed here may also reflect changes in membrane physical properties, including membrane fluidity and lipid-channel interactions, which are themselves highly temperature-sensitive [28–30]. Alterations in membrane viscosity can influence conformational mobility and gating energetics of voltage-gated ion channels. However, the strongly non-uniform and transition-specific temperature dependence observed in the present study, together with the ability of the V416R mutation to largely occlude the temperature-dependent functional inversion, argue that intrinsic gating transitions of Ca_v_3.2 channels represent a major determinant of the observed phenotype.

A major finding of the present study is that the functional consequences of cooling become fundamentally different during repetitive activity compared to conventional voltage-step recordings. Under low-frequency stimulation, Ca_v_3.2-mediated calcium influx largely reflected the expected temperature dependence of macroscopic current amplitude, with enhanced calcium entry at higher temperature and reduced influx during cooling. However, increasing stimulation frequency progressively shifted channel behavior toward sustained calcium influx at lower temperatures. At high frequencies, slowing of inactivation and deactivation kinetics during cooling appeared to promote prolonged channel availability and increased temporal summation of calcium entry during repetitive firing. Thus, although cooling reduced peak current amplitude, the cumulative functional output of Ca_v_3.2 channels during repetitive activity became paradoxically enhanced. These findings indicate that conventional voltage-step protocols incompletely predict Ca_v_3.2 channel behavior under physiologically relevant firing conditions and emphasize the importance of considering gating kinetics in dynamic operational contexts.

Our findings should also be interpreted in light of the properties of the V416R variant used as a mechanistic tool. Although V416R reproduced several aspects of the low-temperature phenotype, particularly the marked slowing of activation, inactivation, deactivation, and recovery from inactivation, the mutation also produced substantial shifts in voltage-dependent activation and, to a lesser extent, inactivation. Consequently, V416R does not represent a pure kinetic mimic of cooling. Rather, it provides an experimental model in which the channel is intrinsically biased toward a slow-gating operational state. The observation that temperature-dependent functional inversion was markedly attenuated in this background nevertheless supports the conclusion that slowing of gating transitions is a major determinant of the phenomenon. Future studies using kinetic modeling may help disentangle the relative contributions of altered gating kinetics and shifted voltage dependence to the observed effects. Although the present study was not designed as a structure–function investigation, the behavior of the V416R variant may provide insight into the molecular basis of temperature sensitivity in voltage-gated calcium channels. The V416 residue occupies a position homologous to residues implicated in disease-associated gating defects in high-voltage-activated calcium channels. Introduction of a bulky positively charged arginine at this position may alter local conformational transitions that couple voltage-sensor movement to pore opening and closing, thereby stabilizing slow-gating states. The observation that an analogous manipulation reproduces several key features of cooling supports the idea that temperature-dependent functional inversion emerges primarily from altered gating kinetics rather than from changes in voltage dependence alone. Together, these findings strongly support the conclusion that the temperature-dependent inversion observed in WT Ca_v_3.2 channels primarily emerges from progressive slowing of gating transitions during cooling. More broadly, these findings raise the possibility that pathological *CACNA1H* variants altering Ca_v_3.2 gating kinetics [11], as well as regulatory proteins or signaling pathways that modulate Cav3.2 channel behavior [31], may similarly influence the temperature dependence of Ca_v_3.2-mediated calcium signaling during repetitive neuronal activity.

The physiological relevance of this mechanism may be particularly important in peripheral sensory neurons exposed to environmental cooling. Ca_v_3.2 channels are abundantly expressed in dorsal root ganglion and trigeminal ganglion neurons, including nociceptive populations involved in pain signaling [5, 6]. Ca_v_3.2 channels are also known to contribute to neuronal excitability, burst firing, and pathological pain states, including peripheral neuropathic pain. Previous studies have shown that cooling can increase firing activity and calcium signaling in subsets of dorsal root ganglion and trigeminal neurons, including neurons not classically considered dedicated cold sensors [22, 24]. In this context, the mechanism identified here may contribute to enhanced excitability and sustained calcium entry during cooling conditions by promoting prolonged Ca_v_3.2 channel activity during repetitive firing. Such a mechanism could be particularly relevant during pathological cold hypersensitivity or cold allodynia, where repetitive neuronal activity occurs under reduced tissue temperature.

More broadly, our findings suggest that temperature-dependent modulation of ion channel kinetics may fundamentally reshape neuronal signaling in ways that cannot be predicted from conventional steady-state electrophysiological measurements alone. This notion is consistent with previous modeling work showing that temperature-dependent modulation of Ca_v_3 channels strongly influences firing behavior, particularly rebound burst discharge, through effects on activation, inactivation, and recovery kinetics [32]. While that study focused on how temperature-dependent channel properties shape spike generation at the systems level, our results provide direct experimental evidence that cooling-induced slowing of Ca_v_3.2 gating transitions can fundamentally alter channel function during repetitive activity, leading to enhanced cumulative calcium influx despite reduced peak current amplitude. Together, these findings highlight the importance of gating kinetics as a key determinant of temperature-dependent neuronal excitability.

In conclusion, the present study identifies a previously unrecognized temperature-dependent functional inversion of Ca_v_3.2 channels during repetitive activity. While cooling suppresses peak Ca_v_3.2 current amplitude under conventional voltage-step conditions, it simultaneously promotes sustained calcium influx during high-frequency firing through disproportionate slowing of channel gating kinetics. These findings reveal that the functional consequences of temperature on Ca_v_3.2 channel activity are highly context-dependent and suggest a potential mechanism by which cooling may enhance excitability and calcium signaling in peripheral sensory pathways.

## Data Availability

All data supporting the findings of this study are available within the paper.
